# Shuttle Transfer of mRNA Transcripts *via* Extracellular Vesicles From Male Reproductive Tract Cells to the Cumulus–Oocyte Complex in Rabbits (*Oryctolagus cuniculus*)

**DOI:** 10.3389/fvets.2022.816080

**Published:** 2022-03-17

**Authors:** Mosleh M. Abumaghaid, Aaser M. Abdelazim, Tareg M. Belali, Muhanad Alhujaily, Islam M. Saadeldin

**Affiliations:** ^1^Department of Laboratory Medical Sciences, College of Applied Medical Sciences, University of Bisha, Bisha, Saudi Arabia; ^2^Department of Basic Medical Sciences, College of Applied Medical Sciences, University of Bisha, Bisha, Saudi Arabia; ^3^Laboratory of Theriogenology, College of Veterinary Medicine, Chungnam National University, Daejeon, South Korea; ^4^Research Institute of Veterinary Medicine, Chungnam National University, Daejeon, South Korea; ^5^Department of Physiology, Faculty of Veterinary Medicine, Zagazig University, Zagazig, Egypt

**Keywords:** extracellular vesicles, testis, epididymis, prostate, oocyte, rabbit

## Abstract

Semen is known to contain an ovulation-inducing factor (identified as a nerve growth factor, NGF) that shows a significant increase in ovulation after semen deposition in induced ovulatory species. However, the interplay between the male reproductive tract cells and oocyte maturation through messenger RNA (mRNA) cargo is yet to be investigated. Extracellular vesicles (EVs) from the primary culture of rabbit prostate (pEVs), epididymis (eEVs), and testis (tEVs) were isolated to examine their contents for several mRNA transcripts through relative quantitative PCR (RT-qPCR). The expressions of *NGF*, neurotrophin (*NTF3*), vascular endothelial growth factor A (*VEGFA*), A disintegrin and metalloprotease 17 (*ADAM17*), midkine (*MDK*), kisspeptin (*KISS1*), and gonadotrophin-releasing hormone (*GNRH1*) were examined in isolated EVs. EVs were characterized through transmission electron microscopy. EV uptake by cumulus cell culture was confirmed through microscopic detection of PKH26-stained EVs. Furthermore, the effects of pEVs, eEVs, and tEVs were compared with NGF (10, 20, and 30 ng/ml) supplementation on oocyte *in vitro* maturation (IVM) and transcript expression. *KISS1, NTF3, MDK, ADAM17, GAPDH*, and *ACTB* were detected in all EV types. *GNRH1* was detected in tEVs. *NGF* was detected in pEVs, whereas *VEGFA* was detected in eEVs. pEVs, eEVs, and 20 ng/ml NGF showed the highest grade of cumulus expansion, followed by tEVs and 10 ng/ml NGF. Control groups and 30 ng/ml NGF showed the least grade of cumulus expansion. Similarly, first polar body (PB) extrusion was significantly increased in oocytes matured with eEVs, pEVs, tEVs, NGF20 (20 ng/ml NGF), NGF10 (10 ng/ml NGF), control, and NGF30 (30 ng/ml NGF). Additionally, the expression of *NGFR* showed a 1.5-fold increase in cumulus cells supplemented with eEVs compared with the control group, while the expression of *PTGS2* (*COX2*) and NTRK showed 3-fold and 5-fold increase in NGF20-supplemented cumulus-oocyte complexes (COCs), respectively. Oocyte *PMP15* expression showed a 1.8-fold increase in IVM medium supplemented with eEVs. Additionally, oocyte *NGFR* and *NTRK* expressions were drastically increased in IVM medium supplemented with pEVS (3.2- and 1.6-fold, respectively) and tEVs (4- and 1.7-fold, respectively). This is the first report to examine the presence of mRNA cargo in the EVs of male rabbit reproductive tract cells that provides a model for the stimulation of female rabbits after semen deposition.

## Introduction

Mammalian ovulation is differently regulated in different species. Several species ovulate on a schedule, irrespective of sexual activity. Others, like rabbits and camels, ovulate strictly after mating with males. Semen is known to contain a neurotrophic factor or an ovulation-inducing factor (identified as a nerve growth factor, NGF) that shows a significant increase in the ovulation after semen deposition in induced ovulatory species (1–5). However, the interplay between the male reproductive tract cells and oocyte maturation through messenger RNA (mRNA) cargo requires elucidation.

Recently, several studies have shown that extracellular vesicles (EVs) can act as mediators of reproductive physiology in males (6–9). EVs are tiny vesicles including exosomes and microvesicles and are secreted by many cells that carry nucleic acid and protein cargo to help cell-to-cell communication; they play an important crucial role in reproduction physiology (10–12).

Semen is one of the biofluids that contain abundant amounts of EVs. Seminal EVs contain high amounts of proteins, lipids, and RNAs. The EVs present in semen could include epididymosomes and prostasomes (13). The majority of proteins are transferred to the sperm subcellular domains and are involved in motility, fertilizing ability, and in protecting sperm against oxidative stress. In comparison, proteins associated with prostasomes regulate capacitation timing. Seminal EVs include a variety of proteins such as enzymes, transporters, chaperones, and many structural proteins. Proteomic studies revealed about 555,438 proteins isolated from the caput and cauda of bull epididymis (14). About 560 proteins of seminal exosomal origin have a role in modulating gene expression through the regulation of DNA methylation, post-transitional histone modifications, and non-coding RNA synthesis (15, 16). Another significance of EVs is its microRNA (miRNA) content. More than 1645 miRNA sequences with miRNA moieties from the let-7, miR-200, miR-26a, miR-103, and miR-191 families have been identified to have a great role in male fertility (7).

This enables these EVs to be involved in post-testicular sperm maturation (17) and to be used as excellent biomarkers in diagnosing early male infertility (18, 19). Studies have revealed that sperm still receive EVs even after ejaculation. These EVs help in sperm motility and capacitation (20) and the whole exchange of spermatozoa after leaving seminiferous tubules (21). Seminal EVs could also help ameliorate erectile dysfunctions (22), while amniotic fluid-derived EVs were reported to be involved in the motivation of spermatogenesis (23). EVs could also be involved in a series of processes starting from gametogenesis, fertilization, and then ending into implantation in physiological and pathological conditions (24). Seminal EVs could also be a good diagnostic tool in assisted reproductive techniques (25). The matter opens the field for its development and improves overall reproductive medicine (26). EVs play a great role in the maintenance of sperm function in epididymis (27) and in the assessment and diagnosis disease-related infertility. It could also be used in the antiretroviral field to identify novel treatments for HIV-1 infection and transmission (28–30).

EVs from seminal fluids often affect semen quality and sperm function, but they have more functions that also extend to the female reproductive physiology. Seminal EVs could interact with the female tract to pose an immune response, which helps in pregnancy and outcomes. These EVs motivate embryo implantation and placental development by promoting leukocyte recruitment and presenting T cells that suppress inflammation and help in embryo implantation (31). The journey of sperms inside the female reproductive tract is not a simple one. A very restricted number of sperms could only be permitted to reach the fertilization site to perform their functions and fertilization. During this journey, sperms continually interact with site-specific maternal components. One of these components is the EVs that play a pivotal role in sperm survival and help it to perform fertilization (32). This illustrates why external EVs from different origins could improve pregnancy outcomes (33) and could also be used to diagnose pregnancy disorders (34).

The lack of studies on the role of EVs in induced ovulatory animal reproduction—such as in rabbits in the current work—motivated the need for unraveling the molecular contents of EVs that can be a possible way for male-to-female interaction. Therefore, the current study aimed to isolate EVs from male reproductive tract cells, analyze their mRNA cargo, and investigate their effects on *in vitro* oocyte maturation.

## Materials and Methods

### Chemicals and Reagents

All chemicals and reagents were obtained from Sigma-Aldrich (St. Louis, MO, USA), unless otherwise specified.

### Tissue Sampling and Culture

Five adult male Chinchilla rabbits (14 months old, weighing about 3 kg) were used for tissue sampling after euthanasia by slaughtering. The lower abdomen was dissected with a sterile scissor and the reproductive tract, including the testicles, was extracted. The prostate, testis, and epididymis (Supplementary Figure 1) were isolated and washed three times with aseptic saline (0.9% NaCl) and transferred into dissecting culture dishes (Falcon, BD Biosciences, Franklin Lakes, NJ, USA). Brain samples were sampled and kept at −80°C until used for RNA extraction as the reference tissue. Brain tissue was used as a positive control reference for the expression of neurotrophic factors. Tissues were dissected with sterile blades and cut into small 1-mm pieces. The tissues were washed three times with phosphate-buffered saline (PBS) supplemented with 5 × penicillin/streptomycin through centrifugation at 1,000 × *g* for 2 min. Then, the tissues were washed two times with the tissue culture medium, distributed into a 60-mm tissue culture dish, and incubated at 38°C, 5% CO_2_ under humid air (Thermo Fisher Scientific, Waltham, MA, USA). Tissue explants were checked daily for attachment, and the culture medium was changed with a freshly prepared medium after explant attachment on day 3 of culture. The tissue culture medium comprised Dulbecco's modified Eagle's medium (DMEM) containing 10% fetal bovine serum (FBS) and 1 × antibiotics. Outgrowths were observed on days 9 and 10, and the remaining explants were removed to establish a monolayer for 4–5 days until 70–80% confluence was reached.

### Isolation and Characterization of Extracellular Vesicles

Prior to EV isolation, the cells were cultured in a DMEM without FBS supplementation for 24 h. Conditioned media were collected, and the EVs were isolated from FBS-free culture medium using Pure Exo Exosomes Isolation kits (101 Bio, Palo Alto, CA, USA). The protein concentration was adjusted to 50 ng/ml using a NanoDrop 2000 (Thermo Scientific™, Waltham, MA, USA). EV pellets were stored at −80°C for further experiments. The resuspended pellets were examined by transmission electron microscopy (TEM) (35). Briefly, 10 μl of the pelleted EV suspension was loaded onto 300-mesh grids, dried, and stained with 2% uranyl acetate. EVs were visualized using an emission TEM (JEM-2100F; JEOL Ltd., Tokyo, Japan) at 120 kV. The mean diameter of the EVs was estimated using ImageJ 1.47t software (National Institutes of Health, Bethesda, MD, USA). Furthermore, the pellets were analyzed using Nanoparticle Tracking Analysis (NTA) version 2.3 build 2.3.5.0033.7-Beta7 (NanoSight, Malvern PANalytical, Salisbury, UK) to determine the particle size and concentration.

### Isolation and *in vitro* Maturation of Cumulus–Oocyte Complexes

Rabbit ovaries were obtained from a local slaughterhouse and transported to the laboratory in 0.9% saline containing 75 mg/ml penicillin and 50 mg/ml streptomycin at 34–36°C. Antral follicles (1–3 mm in diameter) were dissected with sterile blades in 100-mm Petri dishes, washed with Tyrode's albumin lactate pyruvate (TALP) medium, and the cumulus–oocyte complexes (COCs) were isolated under a stereomicroscope (SMZ800, Nikon, Minato-ku, Tokyo, Japan) with zoom range of ×5.0. Dark and homogenous oocytes with at least two layers of compact cumulus cells were selected and washed three times in HEPES-buffered Tyrode's medium (TLH) containing 0.05% (*w*/*v*) polyvinyl alcohol (TLH-PVA). Selected COCs were matured *in vitro* in basic *in vitro* maturation (IVM) medium comprising bicarbonate-buffered tissue culture medium 199 (TCM-199; Gibco, BRL, Grand Island, NY, USA), 0.91 mM sodium pyruvate, 0.57 mM l-cysteine, 10 ng/ml epidermal growth factor, 1 μg/ml insulin, 10 IU/ml human chorionic gonadotropin (hCG), 10 IU/ml equine chorionic gonadotropin (eCG), and 5 μg/ml gentamycin. Approximately 15 COCs were transferred into a four-well dish (Falcon) containing 500 μl of the maturation medium. The COCs were cultured at 38.5°C in 5% CO_2_ in humidified air for 24 h. Cumulus expansion was checked after ending the IVM and was classified into: grade 1, none of the COCs expanded; grade 2, partial COCs expanded; grade 3, COCs were moderately expanded; and grade 4, all COCs were completely expanded with observable intercellular spaces around the oocyte (36). Cumulus cells were removed with gentle pipetting in 0.1% hyaluronidase, and oocytes were selected to examine the first PB extrusion. Denuded oocytes were stained with 10 μg/ml Hoechst 33342 for 10 min and visualized under a fluorescence microscope (Leica DMI4000 B, Leica Microsystems GMS GmbH, Wetzlar, Germany) to detect the nuclear maturation. Oocytes were isolated and kept at −80°C until used for RNA extraction. Cumulus cells were centrifuged at 2,000 × *g* for 2 min and washed two times with PBS, and then the pellets were kept at −80°C until used for RNA extraction. EVs of different origin were supplemented to the IVM medium according to (37). In brief, based on the NTA, the EV pellets were diluted with IVM medium to attain 1.5 × 10^7^ particles/ml. To compare the possible stimulating effects of different EVs on cumulus expansion and oocyte maturation, NGF was supplemented in different doses (10, 20, and 30 ng/ml) to the COC IVM medium and compared with the EVs supplemented with the IVM medium.

### EV Labeling and Uptake by Cumulus Cells

EV pellets were stained with lipophilic fluorescent PKH26 dye according to the manufacturer's instructions. Briefly, 5 μl of EV suspension was diluted in 120 μl of diluent C (provided by the manufacturer), mixed with freshly prepared PKH26 in 125 μl of diluent C to reach a final concentration of 5 × 10^−6^ M, and then incubated for 5 min. Labeling was stopped by the addition of an equal volume of 1% bovine serum albumin (BSA) in PBS and incubation for 1 min. The labeled EVs were washed two times in DMEM using ultracentrifugation and resuspended in 200 μl of culture medium. Cumulus cells were incubated with the labeled EVs for 22 h (35), washed twice with PBS, and stained with 25 μg ml^−1^ bisbenzamide for 10 min at room temperature. For negative controls, the cells were cultured in a basic IVM medium for 22 h. Cells were washed twice with PBS, covered with a VECTASHIELD HardSet Mounting Medium (Vector Laboratories Inc., Burlingame, CA, USA), and left for 5 min at room temperature. The cellular uptake of labeled EVs was observed under a confocal microscope (LSM780, Zeiss, Oberkochen, Germany).

### Relative Quantitative Polymerase Chain Reaction

Total RNA was extracted from tissues (prostate, testis, and epididymis), EVs, oocytes, and cumulus cells using the PureLink RNA Mini Kit (Invitrogen, Carlsbad, CA, USA) according to the manufacturer's protocol. RNA purity and quality were examined with a Nanodrop 2000 spectrophotometer (Thermo Fisher, Waltham, MA, USA). Complementary DNA (cDNA) was synthesized using the High-Capacity cDNA Reverse Transcription Kit (Applied Biosystems, Carlsbad, CA, USA) with a total volume of 20 μl (4 μl of 5 × RT buffer, 1 μl of RT enzyme mix, 1 μl of oligo dT primer, 1 μg of RNA, and up to 20 μl of RNase-free dH_2_O). The expression levels of the transcripts of the examined genes were quantified by relative quantitative real-time PCR (RT-qPCR) through the Applied Biosystem 7500 Real-Time PCR machine and software (Applied Biosystems) with 2X Power SYBR Green PCR Master Mix (Applied Biosystems). The primers are presented in Table 1. The thermal cycling conditions were 95°C for 1 min, followed by 40 PCR cycles for 5 s at 95°C for DNA denaturation and 30 s at 60°C for primer annealing and extension. The melting curve protocol ranged from 65°C to 95°C. Transcripts of the examined genes were quantified in triplicate and calculated as a relative expression to the levels of the reference genes *GAPDH* and *ACTB* in the same sample using the 2^−ΔΔCt^ method (51, 52). The specificity of the RT-qPCR assay was confirmed by both single peaks in the melt curves and by gel electrophoresis. Negative control and cDNA-exempted samples were also used to determine the specificity of real-time reactions. Approximately 20 oocytes per group were processed for each replicate. Experiments were repeated at least three times.

**Table 1:** Primers used for relative quantitative PCR (RT-qPCR) analyses and the roles of these genes in the female reproductive tract.

**Gene name**	**Forward (5^′^ → 3^′^)**	**Reverse (5^′^ → 3^′^)**	**Size (bp)**	**Accession no**.	**Role in the female reproductive system**
*ACTB*	TGGCGCTTTTGACTCAGGAT	TCCGTCACATGGCATCTCAC	177	NM_001101683.1	Housekeeping gene
*ADAM17*	TTCTCCCCGAGCAAACAGTC	CATCAGGATCGTGCTCTGCT	194	XM_002722480.3	Granulosa cell luteinization and oocyte maturation (38–40)
*BMP15*	CCATGGTGAGGCTGGTAAGG	GGTGGAGTTGATGGCGGTAA	150	NM_001199117.1	Follicle development and oocyte maturation (41)
*GAPDH*	AGCTGGTCATCAACGGGAAG	GAAGACGCCAGTGGATTCCA	110	NM_001082253.1	Housekeeping gene
*GDF9*	GCGTTGGAGTCTGAGGTTGA	TCCCCTCCTTGGTAGCGTAA	190	NM_001171350.1	Follicle development and oocyte maturation (41)
*GNRH1*	GCTGCTGACTTTATGCGTGG	CTGGAGGATGGTGCATTCGA	166	XM_008249443.2	Oocyte maturation (42)
*KISS1*	TGTCCTGCGGCAATAAAGGT	TGAAGAAGTCGGACGTGCAG	150	XM_008268539.2	Oocyte maturation (43)
*MDK*	AAGGAGTTTGGAGCGGACTG	CTTTGGCCTTGGCTTTGGTC	175	NM_001082167.1	Oocyte maturation (44)
*MOS*	AAGTCATCTACGGGGCTCCT	GACATCTCGCTCGCTGATCA	197	XM_002710483.1	Meiosis (45)
*NTF3*	TCGCTTATCTCCGTGGCATC	CTTAACGTCCACCACCTGCT	140	XM_017343394.1	Follicle development and oocyte maturation (46)
*NGF*	AGTTCTGGCCTGTGATCGTG	TCCGCCTGTATGCCAATCAG	192	XM_017345952.1	Ovulation and oocyte maturation (47)
*NTRK1*	GTTTCCTTCTCGCCAGTGGA	GGACGAGAAGCAGTGTGGAA	139	XM_008264290.2	Receptors to neurotrophins
*NGFR*	TTGTGTGGCCCCTTGTTTCT	CCTCCTTCTCAGTCTCGGGA	199	XM_008271321.2	Receptors to NGF
*PTGS2*	GTCAAAACCGAGGTGTGTGC	CAGACTCCCTTGAAGTGCGT	178	NM_001082388.1	Cumulus expansion and oocyte maturation (48)
*VEGFA*	CGAGGAGTTCAACGTCACCA	TACCGGGATTTCTTGCGCTT	189	XM_017345155.1	Oocyte maturation (49, 50)

*ACTB, beta actin; ADAM17, A disintegrin and metallopeptidase domain 17; BMP15, bone morphogenetic protein 15; GAPDH, glyceraldehyde-3-phosphate dehydrogenase; GDF9, growth differentiation factor 9; GNRH1, gonadotrophin-releasing hormone; MDK, midkine; MOS, MOS proto-oncogene; NTF3, neurotrophin 3; NGF, nerve growth factor; NTRK1, neurotrophic receptor tyrosine kinase 1; NGFR, nerve growth factor receptor (P75NTR); PTGS2, prostaglandin endoperoxide synthase 2 or cyclooxygenase-2; VEGFA, vascular endothelial growth factor A*.

### Statistical Analysis

Each experiment was repeated at least five times. Cumulus grades, oocyte maturation, and the transcript expression values were presented as the mean ± SEM. Percentages were transformed into arcsine values before analysis. Homogeneity of data variances was analyzed using Levene's test, while normality of the data was verified using the Shapiro–Wilk test. Values were analyzed using one-way analysis of variance (ANOVA). Significant differences among groups were determined using Tukey's honestly significant difference (HSD) test and set at *p* < 0.05.

## Results

### Primary Culture of Prostate, Testis, and Epididymis

**Figure 1:**
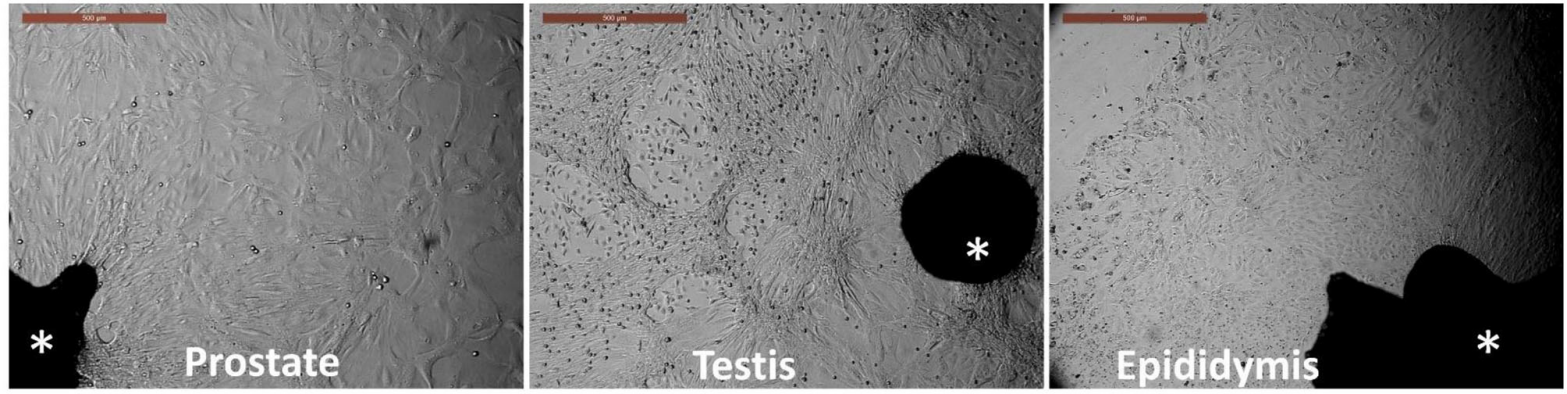
Primary culture of prostate, testis, and epididymis tissues. *Asterisk* indicates the tissue explants. Outgrowths and cell monolayers were maintained until the 14th day of culture. *Scale bar*, 500 μm.

Tissue explants adhered to the culture dishes within 3–4 days, and outgrowths became evident and expanded by day 7 of culture. The culture medium was changed with a fresh medium after tissue adherence until reaching colonies or monolayers of primary outgrowths by day 14 of culture. Each tissue exhibited a different growth pattern, as shown in Figure 1. Prostate cells showed epithelial and increased size cells. Testis cells showed a pattern of circling the outgrowths, while the epididymis showed expanded cuboidal epithelial-like cells.

### Isolation and Characterization of EVs

**Figure 2:**
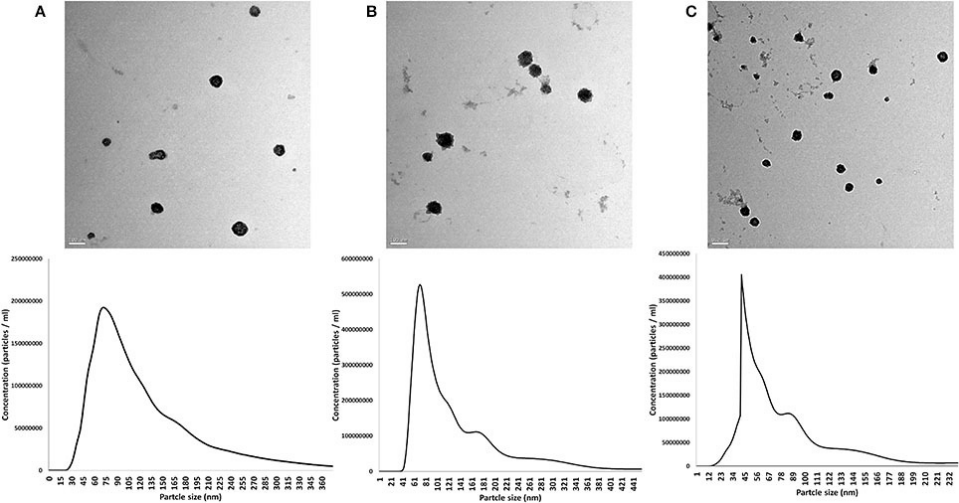
Characterization of extracellular vesicles (EVs) from the rabbit prostate **(A)**, testis **(B)**, and epididymis **(C)**. *Upper panel* shows the TEM images. *Scale bars*, 100 nm for **(A,B)** and 200 nm for **(C)**. *Lower panel* shows the results of nanoparticle tracking analysis (size and concentrations) of the EVs in the corresponding tissue of origin: prostate **(A)**, testis **(B)**, and epididymis **(C)**.

EVs from the prostate, testis, and epididymis were visualized and characterized. TEM images showed the presence of membrane-enveloped vesicles in the conditioned medium of the tissue culture. Furthermore, NTA showed that the mean particle sizes of the isolated EVs were 111.9 ± 7.1, 106.4 ± 8.4, and 108.6 ± 7.3 nm for the prostate-, testis-, and epididymis-derived EVs, respectively (Figures 2A–C).

### Expression of mRNA Transcripts in EVs Derived From the Prostate, Testis, and Epididymis

EVs from the prostate, testis, and epididymis were analyzed for the presence of selected mRNA transcripts in comparison to the tissue of origin and the brain as a reference organ. The transcripts *GNRH1, KISS1, MDK, NTF3, NGF, ADAM17*, and *VEGFA*, as well as *GAPDH* and *ACTB*, were all expressed in the prostate, testis, and epididymis tissues, while *ADAM17* was not detected in the brain samples (Table 2). Prostate-derived EVs (pEVs) showed no expression of the *GNRH1* and *VEGFA* transcripts. Testis-derived EVs (tEVs) showed no expression of the *NGF* and *VEGFA* transcripts. Epididymis-derived EVs (eEVs) showed no expression of the *GNRH1* and *NGF* transcripts (Table 2).

**Table 2:** Expression of the mRNA transcripts in different parts of male cells and their derived extracellular vesicles (EVs).

**Gene name**	**Prostate**	**pEVs**	**Testis**	**tEVs**	**Epididymis**	**eEVs**	**Brain**
*GAPDH*	+	+	+	+	+	+	+
*ACTB*	+	+	+	+	+	+	+
*GNRH1*	+	–	+	+	+	–	+
*KISS1*	+	+	+	+	+	+	+
*MDK*	+	+	+	+	+	+	+
*NTF3*	+	+	+	+	+	+	+
*NGF*	+	+	+	–	+	–	+
*ADAM17*	+	+	+	+	+	+	–
*VEGFA*	+	–	+	–	+	+	+

*+, expression is positive by PCR; –, negative expression by PCR; pEVs, prostate-derived EVs; tEVs, testis-derived EVs; eEVs, epididymis-derived EVs*.

### Effects on Cumulus Expansion and Oocyte Maturation

**Figure 3:**
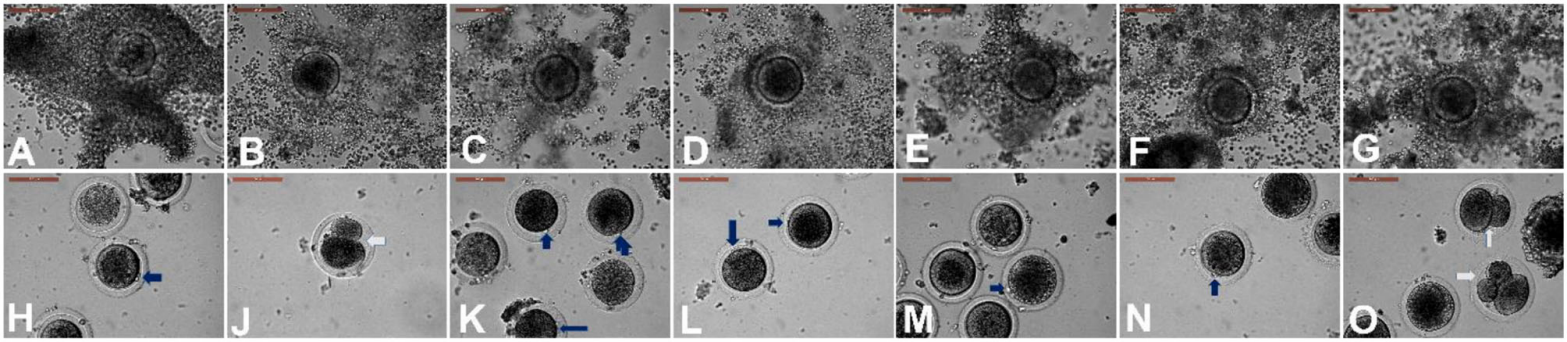
Different degrees of cumulus expansion **(A–G)** and oocyte maturation **(H–O)** after *in vitro* maturation with or without extracellular vesicles (EV) supplementation. **(A)** Control with no expansion. **(B)** Prostate-derived EVs (pEVs) with transparency of intercellular spaces between the cumulus cells (grade 4). **(C)** Testis-derived EVs (tEVs). **(D)** Epididymis-derived EVs (eEVs). **(E–G)** Supplementation with 10 ng/ml NGF **(E)**, 20 ng/ml NGF **(F)**, and 30 ng/ml NGF **(G)**. Panels **(H–O)** show the state of oocyte maturation of the corresponding groups of **(A–G)**. *Blue arrows* indicate the extrusion of the first polar body. *White arrows* indicate spontaneously cleaved oocytes. *Scale bar*, 100 μm.

**Figure 4:**
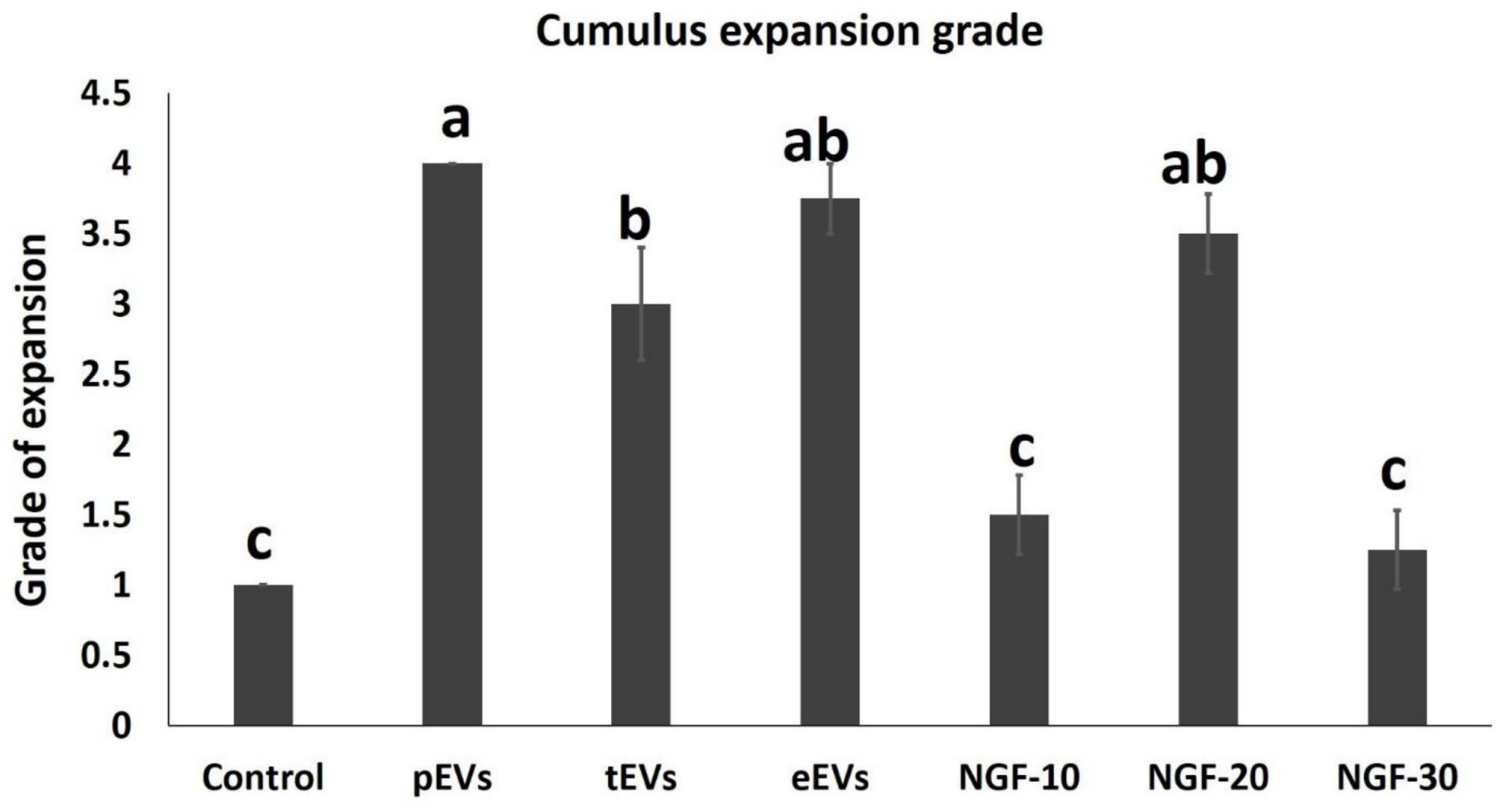
Comparison between the values of the different degrees of cumulus expansion in experimental groups. *Different letters* (*a*–*c*) indicate a significant difference at *p* < 0.05.

**Figure 5:**
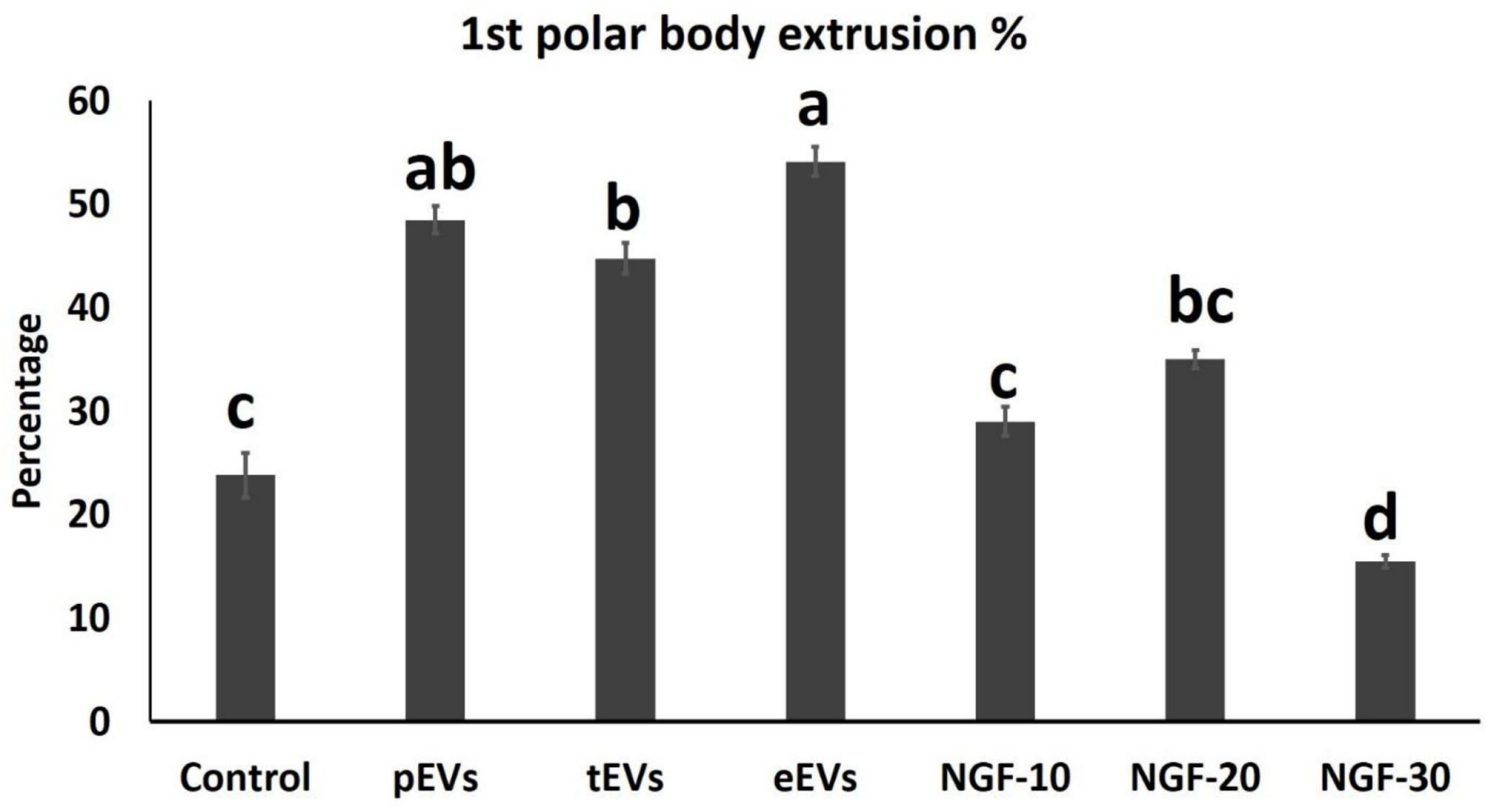
Comparison between the first polar body extrusion values in the oocytes from different experimental groups. *Different letters* indicate a significant difference at *p* < 0.05.

EV supplementation improved the cumulus expansion grades after IVM for 44 h. In the NGF20- (20 ng/ml NGF), pEV-, and eEV-supplemented groups, the space between the cumulus cells was clearly visible, indicating the highest grade of cumulus expansion. The tEV-supplemented group showed moderate expansion when compared to the other NGF-supplemented groups and the control group (Figures 3A–G, 4). Similar effects were observed on the completion of meiosis I, nuclear oocyte maturation, and extrusion of the first PB. Considering that the IVM medium was a defined medium, the control, NGF10 (10 ng/ml NGF), and NGF30 (30 ng/ml NGF) groups showed the least proportion of first PB extrusion compared with the NGF20- (20 ng/ml NGF) and EV-supplemented groups, while the latter groups showed the highest values (Figures 3H–O, 5). Surprisingly, spontaneous cleavage of the oocytes was observed in the pEV-supplemented and NGF30 groups by the end of the 44-h IVM duration (*n* = 5 and *n* = 7, respectively) (Figures 3J,O, respectively).

### EV Uptake by Cumulus Cells and the Effects on Transcript Expression of Cumulus Cells and Oocytes

**Figure 6:**
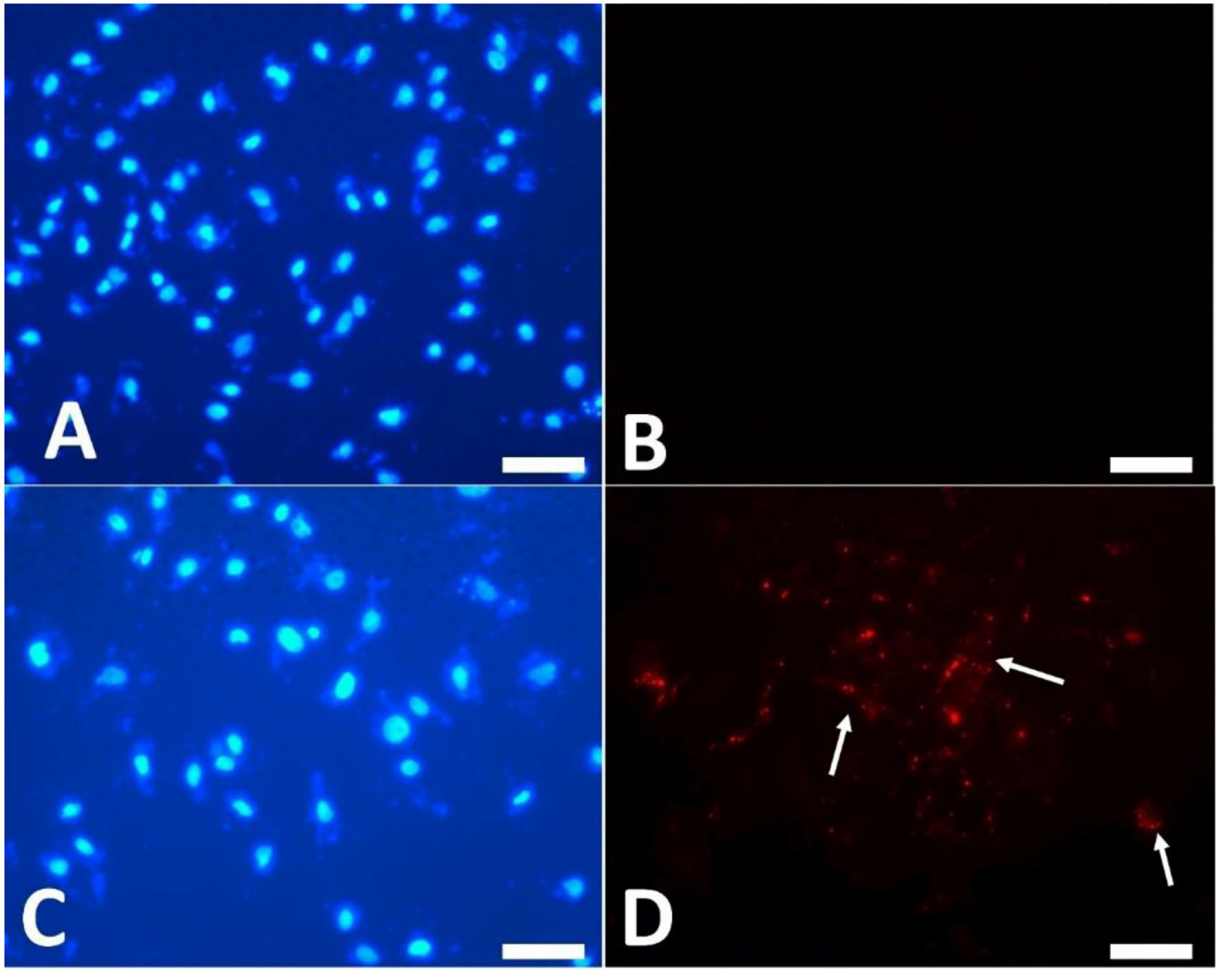
Uptake of PKH26-labeled extracellular vesicles (EVs) by the cultured cumulus cells. **(A,B)** Control cells cultured in plain culture medium. **(C,D)** PKH26 dye was applied to the EVs and kept in the culture medium for 22 h. **(A,C)** Nuclear staining by DAPI. **(B,D)** Localization of red fluorescence staining of EVs (*arrows*). *Scar bar*, 10 μm.

**Figure 7:**
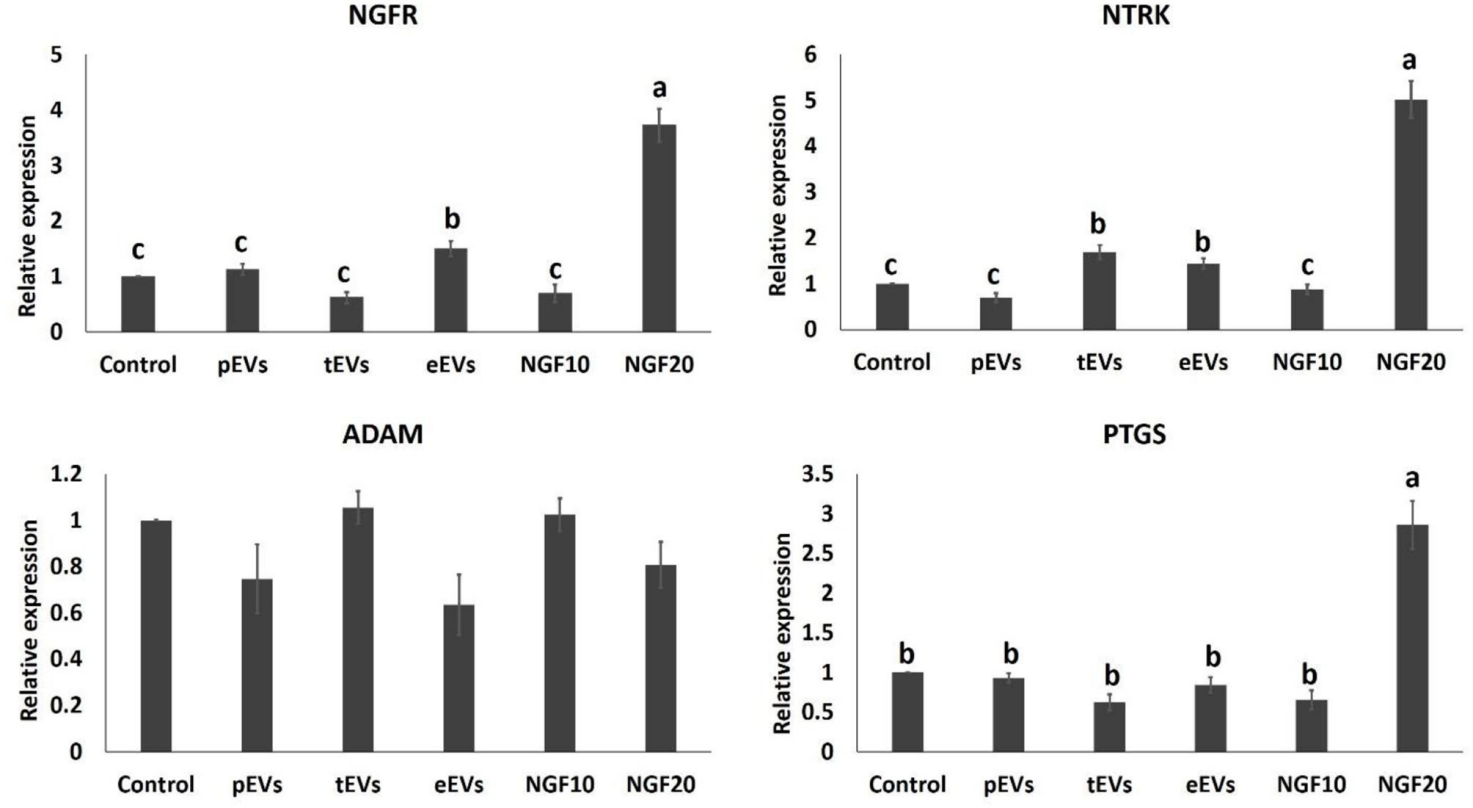
Effects of the prostate, testis, and epididymis extracellular vesicles (EVs) on the gene expression of cumulus cells in comparison to different doses of nerve growth factor (NGF). *Different letters* (*a*–*c*) indicate significant difference at *p* < 0.05.

**Figure 8:**
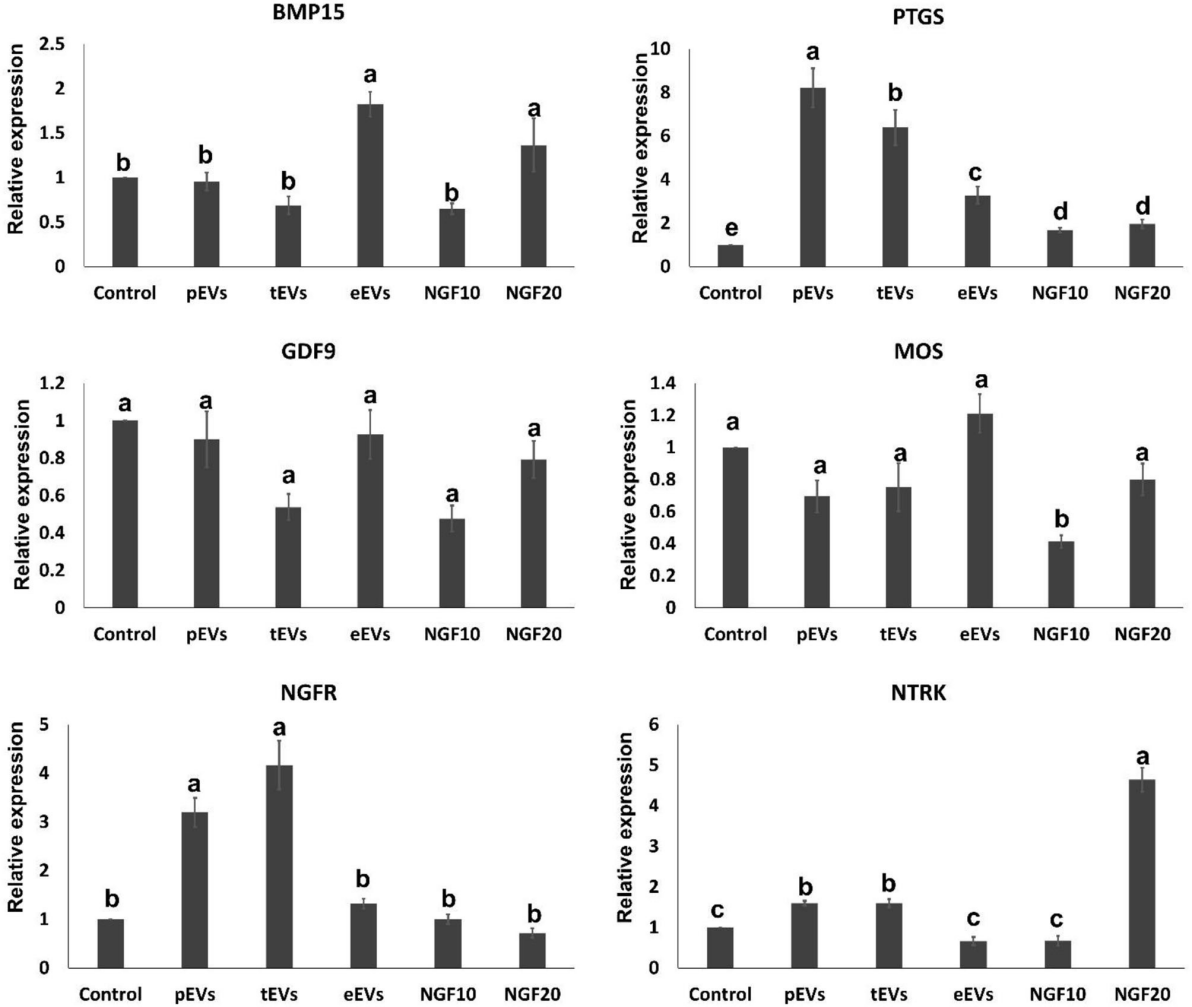
Effects of the prostate, testis, and epididymis extracellular vesicles (EVs) on oocyte gene expression compared to different doses of nerve growth factor (NGF). *Different letters* (*a*–*c*) indicate significant difference at *p* < 0.05.

To examine the EV uptake, the isolated EVs from the epididymis (eEVs) were stained with PKH26 dye and incubated with cultured cumulus cells for 22 h. The results showed red fluorescence signals in the cumulus cells, indicating that EVs were successfully engulfed by the cumulus cells (Figure 6). Furthermore, the expressions of *NGFR* and *NTRK* were significantly increased in the cumulus cells supplemented with the eEVs (Figure 7) compared with the control group. Moreover, the expression of *NTRK* was significantly increased in pEV-supplemented cumulus cells when compared with the control group. *NGFR, NTRK*, and *PTGS* were all increased in the NGF20 group. *ADAM* expression showed no difference among the studied groups (Figure 7). Analysis of the transcript expression in the oocytes showed an increase in the expression of oocyte secreted factors *BMP15* and *PTGS* compared with the control group. The expressions of *PTGS, NGFR*, and *NTRK* also showed a significant increase in the pEV- and tEV-supplemented groups (Figure 8). On the other hand, oocyte secreted factor *GDF9* was decreased in the NGF10 group.

## Discussion

The current results provide for the first time the possible transfer of the mRNA transcript cargo from male cells to female cells *via* EVs secreted from the prostate, testis, and epididymis. EVs were uptaken by cumulus cells and affected the mRNA expression in both cumulus cells and oocytes. These data can explain that the act of ovulation induction in rabbits may depend on some neurostimulants, such as prostate NGF, and other signals, such as *KISS1, MDK*, and *NTF3*, as well as testicular *GNRH1* after transfer of their mRNA transcripts and further translation into proteins/peptides in female reproductive tract cells (53, 54). We also speculate the systemic or endocrine transfer of EVs to affect other organs such as the brain, but further investigations are needed.

The involvement of EVs in shuttling mRNA between cells has been clearly illustrated initially by Valadi et al. (55). Thereafter, several research reports have shown the transfer of mRNA between different kinds of cells, including the embryos (35).

The provided data showed the transfer of the *ADAM17, KISS1, MDK*, and *NTF3* transcripts in the EVs secreted by the prostate, testis, and epididymis. The transfer of these transcripts can improve oocyte maturation and cumulus expansion. *ADMA17* can be involved in granulosa cell luteinization and oocyte maturation (38). Moreover, *KISS1* is involved in regulating the gonadotrope axis and the initiation of puberty, timing control of puberty, and regulation of fertility in adulthood mediated by positive and negative feedback regulation on the hypothalamic–pituitary–gonadal (HPG) axis by gonadal steroids (56). Recently, the protein has been detected to be modulated by leptin and has a role in gonadotropins release (57). Interestingly, *KISS1* and its receptor have been detected in the corpus luteum of pseudopregnant rabbits and showed a luteotropic action by downregulating *PTGS2* expression, which decreased PGF2α and increased PGE2 and progesterone secretion (58). In addition, *KISS1* enhances the oocyte IVM (43). *MDK* is a heparin-binding growth factor expressed abundantly in ovarian follicles and promotes the cytoplasmic maturation of oocytes (44, 59). *NTF3* is a member of the NGF family involved in tissue development and stimulates Leydig cell proliferation (60). Also, it has been involved in the direct control of ovarian function, including follicle assembly, activation of the primordial follicles, follicular growth and development, oocyte maturation, steroidogenesis, ovulation, and corpus luteum formation (46).

However, *NGF* expression was found only in prostate-derived EVs. Elegantly, previous works have revealed the abundance of *NGF* expression in the prostate when compared with other parts of the male reproductive tract, such as the testis, epididymis, bulbourethral gland, and seminal vesicles (61, 62). NGF is a protein involved in the growth of nerves (63, 64) and is expressed in Sertoli cells (65). Also, it could relieve spermatogenesis in azoospermic mice (66) and exerts stimulatory effects on the sperm functions (67, 68). It has a great role in the sustain release of luteinizing hormone (LH), which promotes testosterone secretion in camels and rabbits (5, 69). It stimulates the release of GnRH and pituitary gonadotropins and, consequently, ovulation (70). It is also involved in ovulation inductions in cats, rabbits, and camels (4, 5, 71). Even though moderate levels of NGF improved oocyte maturation (47, 72, 73), paradoxically, excessive NGF supplementation impaired the bidirectional communication between oocytes and cumulus cells and reduced the oocyte competence (74). Our results also showed that the increased levels of NGF hindered oocyte maturation and caused a spontaneous early parthenogenetic cleavage in the oocytes. A similar effect was observed in the pEV-supplemented group, which might be related to their contents of NGF transcripts. Spontaneous cleavage was not observed in the other experimental groups. When comparing EV *vs*. NGF supplementation, similar effects were found; however, the EV-induced effects were more advantageous over NGF supplementation, which might be dose-dependent and caused harmful impacts with increased concentration (i.e., 30 ng/ml).

Additionally, the *VEGFA* transcript was found only in epididymal EVs. *VEGFA* was shown to be an endothelial growth factor and a regulator of vascular permeability (75). Research showed that the VEGFA/VEGF receptor system plays a vital role in regulating progesterone levels (76) and oocyte maturation (49, 50). On the contrary, *GNRH1* was detected in tEVs. *GNRH1* plays a pivotal role in the physiology of reproduction in mammals (77–80). It controls the regulation of ovarian steroidogenesis, decreases proliferation, and induces the apoptosis of ovarian cells (81). GNRH agonist was found to improve oocyte maturation in rabbit follicle-enclosed oocytes matured *in vitro* (42). Moreover, GNRH receptors have been detected in the corpus luteum of pseudopregnant rabbits, suggesting that GNRH1 regulates progesterone synthesis and luteal functions (82).

Our analysis showed increased expressions of the receptors *NTRK1* and *NGFR* in cumulus cells when supplemented with eEVs, while tEVs increased *NTRK* only. On the oocyte level, pEVs and tEVs significantly increased the expressions of both receptors. Moreover, NGF 20 μg/ml showed a significant increase in oocyte *NTRK* expression. *NTRK1* could be activated by one or more neurotrophic NGFs and signals through these receptors involved in regulating cell survival, proliferation, and the fate of neural precursors (83). *NTRK1* is also important in controlling ovarian development (84). In addition, it has been involved in the development and growth of newborn bovine testicular Sertoli cells (85). In the same manner, *NTRK1* plays a significant role in regulating the bovine oviducts *via* its interaction with gonadotropins (86). Moreover, NGF and its receptor are also potent stimulators of prostaglandin biosynthesis of the rabbit uterus (87), and studies have shown that they are immunolocalized in the rabbit corpus luteum (88). *NGFR* is involved in testis-promoting differentiation and/or maintenance (89). NGF/NGFR signals are also involved in regulating the ovarian cycle (90).

However, cumulus cell expression of the *PTGS2* and *ADAM17* transcripts showed no difference with EV supplementation, while *PTGS* showed a significant increase with NGF supplementation. On the level of oocytes, *PTGS* showed a significant increase with all kinds of EV and NGF supplementation. *PTGS* is an enzyme involved in spermatogenesis and steroidogenesis (91). It has two isoforms, *PTGS1* and *PGTS2*, and is considered the committed enzyme in the conversion of arachidonic acid to prostaglandins; it is also involved in the synthesis of other eicosanoids (92). This enzyme plays an important role in early pregnancy, which includes cumulus expansion, ovulation, fertilization and implantation, and decidualization (48).

To illustrate the improved oocyte maturation, we studied the expressions of *MOS, BMP15*, and *GDF9* in the oocytes. Different patterns were observed after EV supplementation. *BMP15* was increased by eEV supplementation, while *MOS* and *GDF9* showed no differences. MOS, a protein kinase, is expressed and performs its function during meiotic division (G2/M) progression in vertebrate oocytes (45). MOS could activate mitogen-activated protein kinase (MAPK), which is required for oocyte maturation (93). GDF9 and BMP15 are oocyte-secreted proteins. They have a significant role in controlling the ovarian function in females. They both modulate the cell fate of somatic granulosa cells, follicular development and oogenesis, and the quality and developmental competence of oocytes (41, 94).

## Conclusion

The results of this study showed that epididymal EVs, in comparison to other experimental groups, provided a better outcome for oocyte IVM and cumulus cells, and this might be related to their neuropeptide contents (*KISS1, MDK*, and *NTF3*) and *ADAM17*, as well as *VEGFA*. We provided evidence for a novel method of communication between male reproductive tract cells and COCs through shuttle transfer of mRNA transcripts, which would be a model for studying the male reproductive tract-derived factors affecting the female reproductive physiology. Prostate, testis, and epididymis-derived EVs can also be used to support oocyte IVM for *in vitro* embryo production. Further analysis of the actions of the translated proteins and peptides is required to elucidate the mechanisms of action of the male reproductive tract cell-derived EVs on female ovulation and subsequent embryonic development.

## Data Availability Statement

The original contributions presented in the study are included in the article/Supplementary Material, further inquiries can be directed to the corresponding author.

## Ethics Statement

The animal study was reviewed and approved by all experimental procedures and animal care were approved by University of Bisha, approval No. [UNCOM/H-07-BH-0896(06/08)].

## Author Contributions

IS, AA, and MMA: conceptualization, writing—original draft preparation, and writing—review and editing. IS and AA: methodology. IS, AA, TB, MA, and MMA: investigation and data curation. MMA: project administration. All authors contributed to the article and approved the submitted version.

## Funding

This study was supported by the University of Bisha, Research Support Program, Project #48.

## Conflict of Interest

The authors declare that the research was conducted in the absence of any commercial or financial relationships that could be construed as a potential conflict of interest.

## Publisher's Note

All claims expressed in this article are solely those of the authors and do not necessarily represent those of their affiliated organizations, or those of the publisher, the editors and the reviewers. Any product that may be evaluated in this article, or claim that may be made by its manufacturer, is not guaranteed or endorsed by the publisher.
