# Supplementary material for: Shuttle Transfer of mRNA Transcripts via Extracellular Vesicles From Male Reproductive Tract Cells to the Cumulus–Oocyte Complex in Rabbits (Oryctolagus cuniculus)
Source: Front Vet Sci. 2022 Mar 17;9:816080. doi: 10.3389/fvets.2022.816080 (PMC8968341; doi:10.3389/fvets.2022.816080)
Supplement: Supplementary file 1 [file Image_1.pdf]

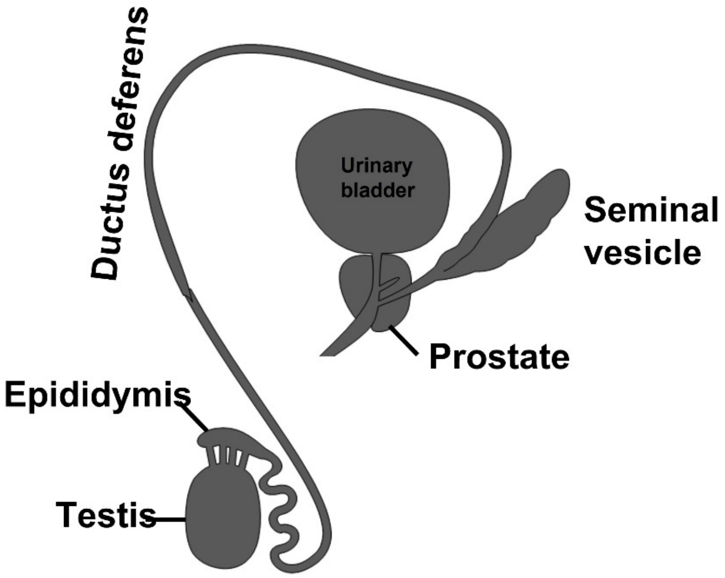

Supplemental Figure 1. Illustrating the reproductive tract of male rabbits and the sites of tissue sampling (prostate, testis, and epididymis).
